# Platelet-Rich Fibrin Low-Speed Centrifugation Protocols and Particulate Bovine Bone on Bone Defects Repair

**DOI:** 10.1590/0103-644020256428

**Published:** 2025-11-21

**Authors:** Lélio Fernando Ferreira Soares, Thaisa Macedo Iunes Carrera, Roberta de Oliveira Alves, Camila Chiérici Marcantonio, Juliana dos Santos Neves, Joni Augusto Cirelli, Guilherme José Pimentel Lopes de Oliveira, Suzane Cristina Pigossi

**Affiliations:** 1 Faculdade de Odontologia da Universidade Federal de Alfenas(UNIFAL/MG), Alfenas, Minas Gerais, Brasil.; 2 Departamento de Periodontia e Implantodontia da Faculdade de Odontologia da Universidade Federal de Uberlândia(UFU), Uberlândia, Minas Gerais, Brasil; 3 Departamento de Diagnóstico e Cirurgia da Faculdade de Odontologia de Araraquara, Universidade Estadual Paulista- UNESP, Araraquara, São Paulo, Brasil

**Keywords:** Bone Regeneration, Bone Substitutes, Growth Factors, Platelet-Rich Fibrin, Preclinical study

## Abstract

Platelet-rich fibrin (PRF) membranes and injectable PRF (i-PRF) are biological agents used to enhance healing, often combined with deproteinized bovine bone mineral (DBBM) for bone regeneration. This study evaluated low-speed centrifugation protocols for the preparation of PRF membranes and i-PRF combined with DBBM (*sticky bone*, SB) in repairing rat tibial bone defects. Sixty-four rats received tibial defects filled with blood clot (CO), PRF membrane alone (PRF), DBBM alone (BO), or i-PRF associated with DBBM (SB). Micro-computed tomography (μCT), histological analysis, and gene expression of molecular markers were assessed at 15 and 45 days. PRF membranes led to greater early bone formation and elevated *Bglap* expression compared to control (p < 0.05). μCT analysis showed that the BO group had significantly greater cortical bone formation at 15 days than the control group (p < 0.05). Additionally, both BO and SB groups promoted increased cortical bone formation at 45 days (p < 0.05), with SB also enhancing cortical thickness at the defect margins. In the medullary region, both BO and SB exhibited higher bone formation and trabecular number (p < 0.05) after 15 and 45 days. Gene expression analysis revealed higher levels of *Alpl, Bglap*, and *Runx2* in the BO group than in SB and control groups (p < 0.05). In conclusion, PRF membranes improved early bone formation compared to a blood clot. DBBM, whether used alone or in combination with i-PRF, also enhanced bone regeneration compared to control. However, the addition of i-PRF did not improve the regenerative potential of DBBM.

## Resumo

Membranas de fibrina rica em plaquetas (PRF) e PRF injetável (i-PRF) são agentes biológicos utilizados para potencializar a cicatrização, frequentemente associados ao osso bovino desproteinizado (OBD) para promover a regeneração óssea. Este estudo avaliou protocolos de centrifugação em baixa rotação para a obtenção de membranas de PRF e de i-PRF combinado com OBD (*sticky bone*, SB) na reparação de defeitos ósseos tibiais de ratos. Sessenta e quatro ratos receberam defeitos preenchidos com coágulo sanguíneo (CO), membrana de PRF (PRF), OBD isolado (BO) ou SB. Foram realizadas análises por microtomografia computadorizada (μCT), avaliação histológica e expressão gênica de marcadores moleculares nos períodos de 15 e 45 dias. As membranas de PRF promoveram maior formação óssea precoce e aumento da expressão de *Bglap* em comparação ao controle (p < 0,05). A análise por μCT mostrou que o grupo BO apresentou formação óssea cortical significativamente maior aos 15 dias em comparação ao controle (p < 0,05). Além disso, os grupos BO e SB promoveram aumento da formação óssea cortical aos 45 dias (p < 0,05), com o grupo SB também apresentando aumento da espessura cortical nas margens do defeito. Na região medular, tanto BO quanto SB apresentaram maior formação óssea e maior número de trabéculas (p < 0,05) após 15 e 45 dias. A análise de expressão gênica revelou níveis mais elevados de *Alpl*, *Bglap* e *Runx2* no grupo BO em comparação aos grupos SB e controle (p < 0,05). Em conclusão, as membranas de PRF melhoraram a formação óssea precoce em comparação ao coágulo sanguíneo. O OBD, utilizado isoladamente ou em combinação com i-PRF, também promoveu maior regeneração óssea em comparação ao controle. No entanto, a adição de i-PRF não aumentou o potencial regenerativo do OBD.

**Figure:**
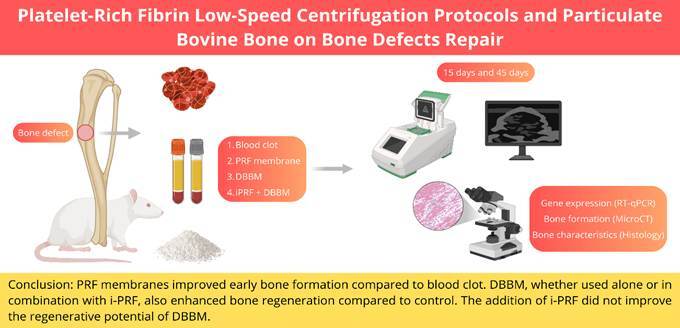

## Introduction

Alveolar ridge preservation (ARP) using a combination of various surgical techniques and biomaterials is considered a valid therapeutic option that significantly reduces post-extraction ridge dimensional changes. ^1^ ARP procedures aim to minimize crestal bone resorption and maximize bone regeneration by immediately placing a biomaterial within the socket. ^2^ Among the biomaterials and biological agents investigated in the literature, using platelet concentrates, associated or not with bone grafts or membranes, offers safe and favorable results. ^3^

Described as the second generation of platelet concentrates, Platelet Rich Fibrin (PRF) is prepared from plasma, after centrifugation of whole blood without the addition of any coagulant. The PRF is formed by slow, spontaneous coagulation, maintaining activated platelets and white blood cells in a fibrin-rich matrix, as in a natural blood clot. ^4^ The gradual and slow release of growth factors, pro- and anti-inflammatory cytokines, and matrix glycoproteins from PRF for at least 7 days ensures long-term healing effects, instilling confidence in its efficacy. ^5^ In this way, PRF acts to improve and accelerate the healing of soft and hard tissues by cell migration/proliferation modulation and immune organization. ^4^

Recent findings have demonstrated that variations in centrifugation protocols significantly influence the fibrin architecture and biological properties of platelet-rich fibrin, thereby altering its clinical efficacy. ^6^ High-speed centrifugation (~700 g) typically concentrates cells at the bottom of the PRF tubes, resulting in a matrix with uneven cell and growth factor distribution. Conversely, low-speed centrifugation (~200 g) promotes the formation of a PRF matrix characterized by a more homogeneous distribution of platelets, leukocytes, and growth factors throughout the fibrin network. ^7^ This low-speed protocol has been associated with higher retention of biologically active components, including a greater concentration of platelets, leukocytes, and key growth factors critical for tissue regeneration and healing. ^6^ Such improvements in PRF composition have been shown to enhance its therapeutic potential, particularly in regenerative procedures, like ARP. ^8^

Similarly, an injectable platelet-rich fibrin (i-PRF) was also developed by decreasing the G-force (700 rpm, 60g for 3 min). This protocol suggests that a greater percentage of cells, including platelets and leukocytes, remain in the upper compartment of the centrifuge tubes where i-PRF is collected, providing more cells and molecules that can assist in tissue regeneration. ^9^ The i-PRF mixed with bone graft granules formed a "sticky bone" material. In these cases, the liquid fibrinogen in i-PRF slowly converts into fibrin, which acts as a binding agent, agglomerating or coating biomaterials. ^10^

Based on the advantages of low-speed PRF protocols, this study aimed to evaluate, in a rat tibial bone defect model, ^1^ the regenerative potential of PRF membranes used alone compared to the blood clot, and ^2^ the effect of injectable PRF combined with a bone graft material, simulating its clinical use as a 'sticky bone' formulation.

## Materials and methods

A detailed description of the sample size calculation, animal care, experimental procedures, and outcome measures used in this study is provided in the Supplementary Material.

### Study design

A total of 64 rats were used in this study [n=16 per group, divided into two time points (15 and 45 days)]. The animals were randomly assigned to four groups according to the type of biomaterial used to fill the defects: CO: blood clot-filled bone defects; PRF: bone defects filled with PRF membranes; BO: bone defects filled with DBBM; and SB: bone defects filled with *Sticky bone* (i-PRF + DBBM).

### Experimental procedures

### PRF obtention

Donor rats were used in the present study to prepare PRF. Approximately 7mL of cardiac blood was collected through direct cardiac puncture of the left ventricle using a 23 g BD Vacutainer® Safety-Lok™ Blood Collection Set scalp, and a 9 mL Vacuette® tube. A blood collection tube without anticoagulants was used to prepare the i-PRF, and a tube with a siliconized clot activator was used to prepare the PRF membranes. The whole blood was immediately centrifuged (Centrifuge FibrinFuge25®, Montserrat, China) after collection.

PRF membranes were obtained after centrifugation at 1659 rpm for 10 min (200 g). ^11^ After centrifugation, the upper polymerized layer was removed from the tube using tweezers and separated from the lower portion using scissors. The material was placed in a specific case and compressed for 2 minutes to remove the plasma and form the membrane.

For i-PRF preparation, a low-speed centrifugation and relative centrifugation force (RCF) protocol was applied at 700 rpm for 8 min (44g). ^12^ After centrifugation, the upper layer was aspirated using a syringe, and the i-PRF was agglutinated to the DBBM granules (0.25mm - 1mm; Bio-Oss®, Geistlich AG, Wolhusen, Switzerland) to form the SB. ^10^

Due to the blood collection method, a humane endpoint was applied to the animals after the collection by CO_2_ inhalation.

### Surgical procedure

**Figure 1:**
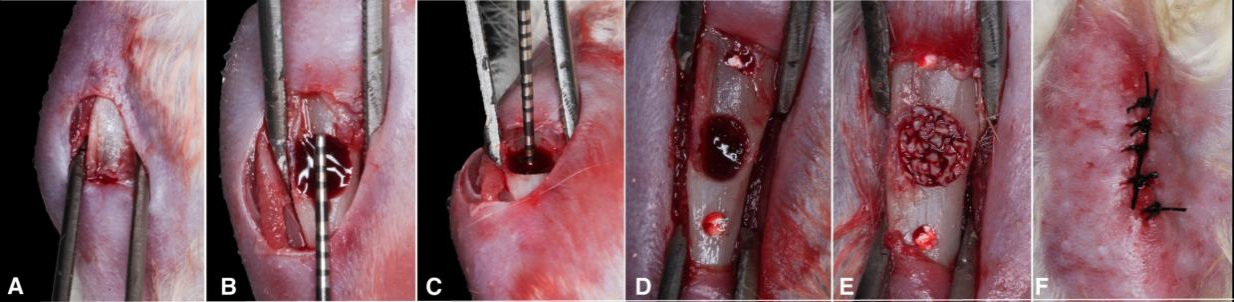
Sequence of surgical defect preparation. A: Exposure of the tibia; B: Circular defect with a diameter of 3.5mm; C: Depth of 1.5mm of the defect created; D: Adjacent markings; E: Defect filled with bone xenograft granules; F: Suture of the animal's cutaneous plane.

Non-critical bone defects presenting 3.5 mm in length and width and 1.5 mm in depth ^13^ ^,^ ^14^ were created in both tibiae using a round burr. The tissue was sutured by planes internally with resorbable wire 5.0 (Vicryl Ethicon, Johnson & Johnson, São José dos Campos, Brazil) and externally with silk thread 4.0 (Ethicon, Johnson & Johnson, São José dos Campos, Brazil) (Figure 1). Animals were euthanized by CO_2_ inhalation after 15 and 45 days.

### Outcome measures

### *Micro-computed tomography (*μ*CT) analysis*

Samples were scanned by a μCT device (Skyscan 1272, Aatselaar, Belgium) with the following parameters: pixel size of the camera, 7.4 μm; power of the X-ray tube, 65 kVp; X-ray intensity, 385 μA; integration time, 300 ms; filter, Al-1 mm; and voxel size, 18 μm. The images were reconstructed, spatially repositioned, and analyzed by specific software (NRecon, Data Viewer, CTAnalyser, Aatselaar, Belgium).

**Figure 2:**
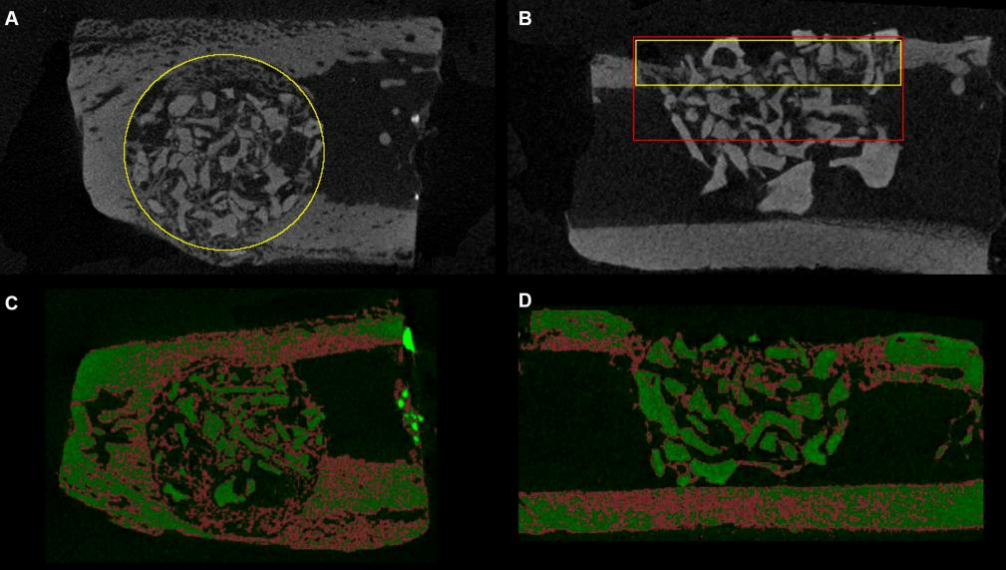
Regions of interest and threshold defined for bone micro-structural parameters analysis by micro-computed tomography. A: Circular ROI defined around the hole defect; B: ROIs including both cortical (yellow) and medullary areas (red); C: Representative threshold applied to transaxial images; D: Representative threshold applied to coronal images.

Two different regions of interest (ROI) were analyzed: the circular ROI 1 corresponded to the total volume of the defect considering only the cortical area (3.5 mm in diameter x 0.54 mm in depth) (Figure 2A); the rectangular ROI 2 corresponded to the total volume of the defect considering both cortical and medullary area (ROI 2: 3.5 mm in diameter x 1.08 mm in depth) (Figure 2B).

The following bone micro-structural parameters of newly formed bone were evaluated at the cortical level (transaxial slices): mineralized new bone tissue volume/tissue volume percentage ratio (BV/TV 1), residual graft particles/tissue volume percentage ratio (BV/TV 2), and average cortical thickness (Ct.Th, mm). ^15^ Ct. Th analysis considered the average cortical thickness of six serial slices from thirty coronal slices in the most central region of the defect. The ROI defined for this analysis included the cortical limits of the defects (coronal slices) (Figure 2B).

To assess the defect’s bone microstructure (trabecular and cortical morphology), both the mineralized new bone tissue volume and the residual graft particles were included in ROI 2 analysis. The analyses included were: BV/TV (%), trabecular thickness (Tb.Th, mm); trabecular separation (Tb.Sp, mm), and trabecular number (Tb.N, mm_−1_) ^15^.

### Descriptive histology

Specimens were prepared with semi-serial 5 μm sections for staining in Hematoxylin & Eosin (HE) for descriptive histology analysis. The sections were analyzed under an optical microscope (DIASTAR - Leica Reichert & Jung products, Germany) with a 2.5/10x magnification objective and 10x magnification eyepieces. They were captured and sent to a microscope using a 10x magnification camera, video DXC-1107A/107AP (Sony Electronics Inc., Japan).

### Bone molecular markers expression (RT-qPCR)

A trephine burr measuring 4.1 mm in diameter (Neodent®, Curitiba, Brazil) was centered in the region of the bone defect to obtain sufficient tissue for gene expression analyses. After tissue collection storage, an RNA extraction by TRIzol (Invitrogen™, Carlsbad, USA) followed by complementary DNA (cDNA) using the OligodT^20^ with the High-Capacity cDNA Reverse Transcription kit (Invitrogen™), according to the manufacturer's instructions.

Real-time PCR (RT-qPCR) reactions were performed using the TaqManTM system (Applied Biosystems, Foster City, USA). The gene for endogenous control of the reaction was glyceraldehyde-3-phosphate dehydrogenase (*GAPDH*, cat. nº Mm99999915_g1), whose expression level was used to normalize the expression of the genes of interest, *Alpl* (Alkaline Phosphatase; cat. nº Mm00475834_m1), *Runx2* (RUNX; cat. no. Mm00501584_m1), *and Bglap* (Osteocalcin; cat. no. Mm00649782_gH). The relative gene expression values of the genes of interest were analyzed by averaging the ΔΔCt values of each gene of interest.

### Statistical analysis

The data were first submitted to the Shapiro-Wilk test to assess normality and the Levene test to evaluate homoscedasticity. Comparisons between the PRF and CO groups were performed using paired *t*-tests to assess both intergroup and intragroup differences. For the CO, BO, and SB groups, a two-way ANOVA followed by Tukey's post hoc test was used to analyze intragroup and intergroup differences in microtomographic data. Gene expression comparisons between the CO and PRF groups were conducted using unpaired t-tests. For the BO, SB, and CO groups, data were analyzed using one-way ANOVA followed by Tukey’s post hoc test. To analyze residual graft particles, paired *t*-tests were used to compare the BO and SB groups at each time point. All statistical analyses were performed using GraphPad Prism 9.5 software (San Diego, USA), with a significance level set at 5% (p < 0.05).

## Results

### μ*CT analysis*

### Main analysis: cortical defect area evaluation

**Figure 3:**
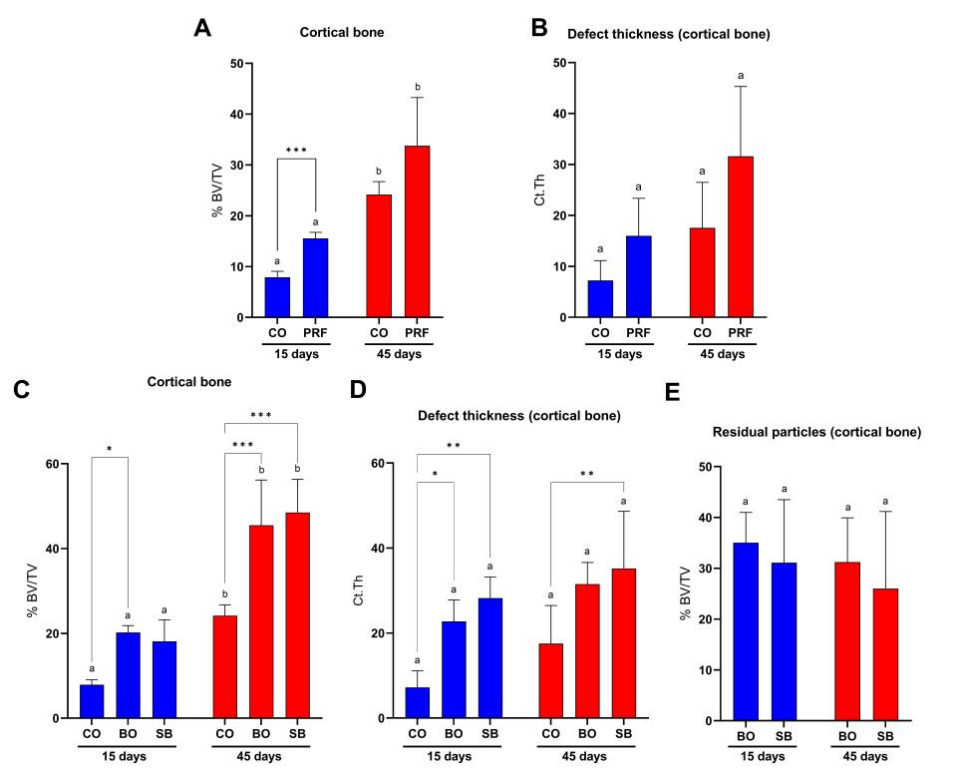
Micro-computed tomography analyses conducted in the cortical defect area. A: Percentage of new bone formation (BV/TV) in CO and PRF groups; B: Cortical bone defect thickness (Ct.Th) in CO and PRF groups; C: Percentage of new bone formation (BV/TV) in CO, BO, and SB groups; D: Cortical bone defect thickness (Ct.Th) in CO, BO, and SB groups; E: Percentage of residual graft particles (BV/TV). Different letters (a, b) indicate intragroup statistical differences (*p* < 0.05); asterisks (*) indicate intergroup statistical differences (*p* < 0.05). CO: Control group; PRF: PRF membrane group; BO: DBBM alone group; SB: Sticky bone group (i-PRF + DBBM).

At 15 days, defects filled with PRF membranes (Figure 3A) and those filled with DBBM (Figure 3C) exhibited a significantly higher percentage of newly formed bone (BV/TV) compared to the control group (*p* < 0.05). By 45 days, although the PRF group showed higher BV/TV values (Figure 3A), only the groups treated with BO and SB maintained significantly elevated BV/TV values relative to the control (p < 0.05) (Figure 3C). Intragroup analysis revealed that all evaluated groups showed a significant increase in BV/TV over time, from 15 to 45 days (*p* < 0.05) (Figure 3A and 3C).

Regarding cortical thickness (Ct.Th), although the PRF group presented higher mean values than the control group at both 15 and 45 days (Figure 3B), significantly greater thickness at 15 days was observed only in the BO and SB groups compared to the control (p < 0.05) (Figure 3D). At 45 days, significant cortical thickness remained only in the SB group relative to the control (p < 0.05) (Figure 3B and 3D).

As for the percentage of residual graft particles (BV/TV), no significant differences were observed between the BO and SB groups in either intergroup or intragroup comparisons (*p* > 0.05) (Figure 2E).

### Secondary analysis: cortical and medullary defect area evaluation

**Figure 4:**
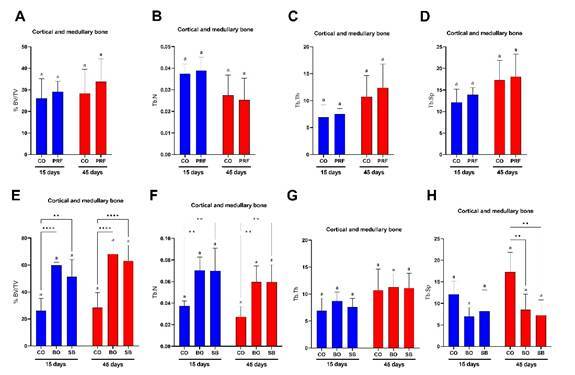
Micro-computed tomography analyses conducted in the cortical and medullary defect area. A: Bone defect filling percentage (bone tissue/biomaterial; BV/TV) in CO and PRF groups; B: Trabecular number (Tb.N) in CO and PRF groups; C: Trabecular thickness (Tb.Th) in CO and PRF groups; D: Trabecular separation (Tb.Sp) in CO and PRF groups; E: Bone defect filling percentage (bone tissue/biomaterial; BV/TV) in CO, BO, and SB groups; F: Trabecular number (Tb.N) in CO, BO, and SB groups; G: Trabecular thickness (Tb.Th) in CO, BO, and SB groups; H: Trabecular separation (Tb.Sp) in CO, BO, and SB groups. Different letters (a, b) indicate intragroup statistical differences (p < 0.05); asterisks (*) indicate intergroup statistical differences (p < 0.05). CO: Control group; PRF: PRF membrane group; BO: DBBM alone group; SB: Sticky bone group (i-PRF + DBBM).

At both 15 and 45 days, no statistically significant differences were observed between defects filled with PRF membranes and the control group for BV/TV, trabecular number (Tb.N), trabecular thickness (Tb.Th), or trabecular separation (Tb.Sp) (p > 0.05) (Figure 4A to 4D). In contrast, the groups with biomaterial addition (BO and SB) exhibited significantly higher BV/TV and Tb. N values at both time points compared to the control group (p < 0.05) (Figure 4E to 4F). No significant intragroup or intergroup differences were found for Tb. Th at either time point (p > 0.05) (Figure 4G). Finally, greater trabecular separation was also observed in the control group compared to the BO and SB groups at 45 days (p < 0.05) (Figure 4H). The addition of i-PRF to DBBM did not result in significant statistical differences in any of the analyses (p > 0.05) (Figure 4E to 4H).

### Descriptive histology

**Figure 5:**
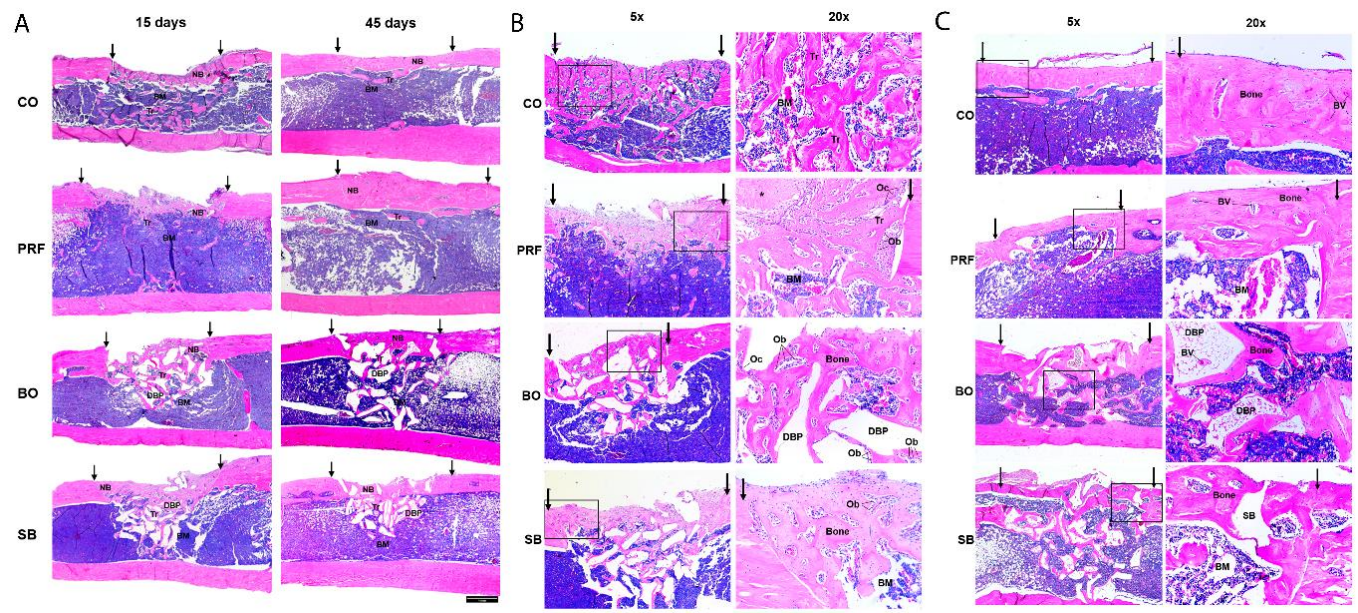
Representative images of histological sections. A: After 15 and 45 days (2.5x); B: After 15 days (5x and 20x). C: After 45 days (5x and 20x). Arrows: Defect edges; Asterisk: Residual PRF membrane; BM: Bone marrow; BV: Blood vessel; DBP: Deproteinized bone particle; NB: New bone; Ob: Osteoblast; Oc: Osteoclast; Tr: Bone trabeculae. CO: Control group; PRF: PRF membrane group; BO: DBBM alone group; SB: Sticky bone group (i-PRF+DBBM).

Figure 5 shows the histological aspect of the tibiae defects after 15 and 45 days. At 15 days post-treatment, newly formed bones were observed in all treatments. Bone trabeculae were immature, rich in osteocytes, and occupied the defect region from edge to edge; however, the volume of scar tissue varied depending on the material used. In general, no inflammatory infiltrate was observed. After 45 days, the defect was completely closed in all groups, although the volume of bone tissue inside the defect varied. The new bone tissue was lamellar (mature), vascularized, and covered by periosteum. More detailed histological analyses for each group are presented in the Supplementary Material.

### Bone molecular markers expression (RT-qPCR)

**Figure 6:**
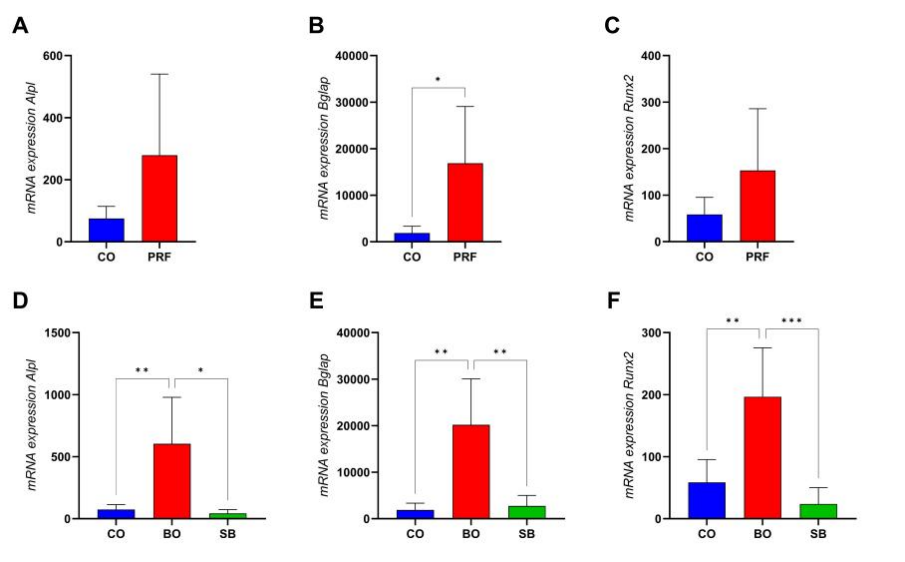
Bone molecular markers expression. A: *Alpl*. B: *Bglap*. C: *Runx2*. *: Intergroup statistical difference (p<0.05). CO: Control group; PRF: PRF membrane group; BO: DBBM alone group; SB: Sticky bone group (i-PRF+DBBM).

Gene expression analysis of *Alpl*, *Bglap*, and *Runx2* was performed at 15 days (Figure 6). Defects filled with PRF membranes showed increased expression of all three markers compared to the control, but a statistically significant difference was observed only for Bglap (p < 0.05) (Figure 6B). Similarly, the isolated use of DBBM showed significantly elevated expression levels of *Alpl*, *Bglap*, and *Runx2* compared to both the group treated with i-PRF combined with biomaterial (SB) and the control group (p < 0.05) (Figure 6D to 6F).

## Discussion

This study investigated the regenerative effects of low-speed centrifugation-derived PRF in two experimental approaches using a rat tibial bone defect model. First, PRF membranes were evaluated alone and compared to blood clot (CO) to assess their independent regenerative potential. Second, i-PRF combined with a bone graft (SB) was compared to both the graft alone (BO) and blood clot (CO) to examine the added benefit of i-PRF in a clinically relevant sticky bone formulation. The findings demonstrated that PRF membranes enhanced early bone formation compared to CO. At the same time, BO and SB promoted greater and more sustained bone regeneration than CO, with no additional benefit from adding i-PRF to the graft.

Microtomographic analysis of the cortical bone revealed that PRF membranes exhibited significantly increased bone formation (BV/TV) at 15 days compared to control, associated with higher *Bglap* gene expression, suggesting an early osteoinductive effect. Previous studies also described superior bone formation in calvaria bone defects filled with standard PRF membranes compared to blood clot ^16^ ^,^ ^17^. To, Su ^18^ showed superior bone formation in dog sockets filled with the Advanced-PRF membrane (200g for 8 min) compared with blood clot.

Because tibial defects are primarily cortical in nature, cortical bone analysis was prioritized in our study; however, a complementary evaluation of the trabecular region was also performed to provide a broader perspective on bone healing. In the cortical and medullary regions, however, the effects of PRF were more limited. PRF membranes did not significantly improve trabecular parameters (BV/TV, Tb.N, Tb.Th, or Tb.Sp) compared with the control, suggesting that their regenerative potential may be confined to early healing phases. ^19^ This limited effect could be related to the significant decrease in growth factor release, such as TGF-β1 and PDGF, after 3, 7, and 14 days, which may not be sufficient to sustain bone regeneration over time. ^20^ Moreover, the histological description of PRF group samples in the present study showed that bone healing occurred along the membrane but not inside the PRF clot. This observation suggests that the dense fibrin network of PRF seems to block cell penetration as described previously by Knapen and Gheldof ^20^.

Overall, both the BO and SB groups showed greater bone formation than the control at the cortical level (excluding DBBM particles) and in the cortical-medullary analysis (including DBBM), with no significant difference between them.** **These results may be explained by the osteoconductive properties of DBBM particles, along with their slow resorption, which provide a scaffold for cell migration and new blood vessel formation during bone regeneration. ^21^**** However, one of the main disadvantages of DBBM grafts is their very slow and often incomplete resorption, which can result in a significant volume of graft material that is not replaced by new bone. ^22^ ****In the present study, both μCT and histological analyses confirmed the presence of residual DBBM particles within the defect area in both BO and SB groups.

Clinicians have proposed the association between i-PRF and DBBM (SB) to enhance wound healing by promoting agglomeration or coating of graft particles. ^23^ In clinical settings, i-PRF can effectively maintain the particulate bone together without the need for an additional membrane for graft particle stabilization. ^24^ Moreover, *in vitro* studies have shown that i-PRF can induce the mobilization and growth of bone regenerative cells ^25^ and significantly increase human gingival fibroblast cell migration, proliferation, and spreading. ^9^ A preclinical study comparing bilateral maxillary sinuses grafted with either DBBM + i-PRF or DBBM alone showed that i-PRF accelerated early vascularization, bone remodeling, and graft substitution. However, it did not improve long-term bone volume. ^26^ In contrast, in the present study, the addition of i-PRF obtained through the low-speed concept to DBBM increased bone thickness. However, it did not significantly enhance overall bone formation compared to DBBM alone. This may reflect the intrinsic regenerative capacity of non-critical defect models, in which complete healing occurs regardless of the biomaterial used, thereby limiting the observable effect. Moreover, the standard i-PRF centrifugation protocol described in the literature is 700 rpm for 3 min (60 g); ^21^ however, in a more recent study, ^12^ an even lower RCF concept is described [700 rpm for 8 min (44 g)]. This low-speed protocol has been reported to yield higher concentrations and greater release of regenerative cells and growth factors in rodent models, justifying its selection for the present investigation.

Bone molecular markers, such as *Alpl*, *Bglap*, and *Runx2* genes, indicate osteoblastic differentiation and bone formation/remodeling. In the present study, RT-qPCR analysis showed that the DBBM and PRF groups exhibited higher biomarker expression than the control group, confirming the osteoinductive potential of these materials reported in a previous study. ^18^ Conversely, the i-PRF application resulted in significantly lower expression of all biomarkers. Our results support previous findings ^27^ showing that the PRF reduced *Runx2* and *Vegfa* gene expression after 14 days. It is hypothesized that the healing process in the PRF-treated group was already advanced, requiring fewer biomarkers. ^28^ Other authors suggest that the growth factors released from PRF matrices are already at the needed level locally and that no additional production would be necessary by the host tissue. ^29^ It is important to emphasize that PCR-based gene expression is a complementary analysis that provides insights into the molecular activity associated with regeneration. However, the primary outcome of this study is cortical bone formation, which more directly reflects the efficacy of the regenerative strategy in this tibial defect model and should be considered the most relevant indicator. Notably, in the present study, the lower expression of bone biomarkers in the SB group did not translate to reduced bone formation at either 15 or 45 days, suggesting no additional effect from the i-PRF + DBBM combination.

It is important to note some limitations of this study. The method used for blood collection, i.e., cardiac puncture, is a non-survival method; therefore, donor rats were used for PRF membrane and i-PRF preparation. Additionally, the effects of i-PRF on gene expression were not investigated beyond 15 days, which limits our ability to determine whether the observed trends persist over time. Protein-level analysis of the investigated growth factors was not performed due to methodological limitations. Moreover, high standard deviations observed in some outcomes may have limited the detection of statistically significant differences. Although increasing the number of animals could help reduce variability and clarify these differences, the number of animals was determined based on a prior sample size calculation and ethical considerations. Another limitation is the absence of a group combining PRF membranes with DBBM, which would have allowed a direct comparison between the two PRF formulations when used with bone graft material. This group was not included because the primary aim was to evaluate the isolated effect of the membrane before suggesting its use in combination with grafts for more challenging defects. On the other hand, i-PRF is commonly used in combination with DBBM, which justifies its inclusion as a combined treatment group in the present study.

Within the limits of this study, PRF membranes improved early bone formation compared to a blood clot. DBBM, whether used alone or in combination with i-PRF, also enhanced bone regeneration compared to control. However, the addition of i-PRF did not improve the regenerative potential of DBBM.
